# Perceptions, Knowledge and Attitudes among Young Adults about Prevention of HPV Infection and Immunization

**DOI:** 10.3390/healthcare10091721

**Published:** 2022-09-08

**Authors:** Maria Sidiropoulou, Georgia Gerogianni, Freideriki Eleni Kourti, Despoina Pappa, Afroditi Zartaloudi, Ioannis Koutelekos, Evangelos Dousis, Nikoletta Margari, Polyxeni Mangoulia, Eftychia Ferentinou, Anna Giga, Michail Zografakis-Sfakianakis, Chrysoula Dafogianni

**Affiliations:** 1 School of Social Sciences, Hellenic Open University, 26335 Patra, Greece; 2 Department of Nursing, University of West Attica, 12243 Athens, Greece; 3 School of Medicine, National and Kapodistrian University of Athens, 10679 Athens, Greece; 4 Department of Nursing, National and Kapodistrian University of Athens, 10679 Athens, Greece; 5 Department of Nursing, Hellenic Mediterranean University, 72100 Crete, Greece

**Keywords:** HPV vaccine, human papilloma virus, attitudes, cervical cancer, prevention, knowledge, perception

## Abstract

Introduction: Human papilloma virus (HPV) is one of the most common sexually transmitted infections and is widely known as the main causative agent for cervical cancer. The aim of this study was to investigate the perceptions, knowledge and attitudes of young Greek adults concerning prevention of HPV infection and HPV immunization. Material and Methods: This constitutes a cross-sectional online survey. A convenience sample of young Greek adults (*n* = 883) residing in Greece, aged 17 to more than 35 years was surveyed from December 2020 to March 2021. Two validated questionnaires were used to collect data. Results: Participants demonstrated moderate knowledge about HPV infection and vaccination, with a mean knowledge score of 53.26 (SD ± 20.65) and 38.92 (SD ± 17.58), respectively. Cronbach’s alpha value was 0.77 and 0.80. Female participants were better informed than males. Approximately 52.3% of respondents had been vaccinated and 65.5% were willing to get vaccinated in the future. Vaccination rate was significantly associated with gender (OR = 11.99; 99% CI = 6.59–21.84), knowledge about the HPV vaccine (OR = 1.04; 99% CI = 1.03–1.04) and age (OR = 0.07; 99% CI = 0.03–0.15). Reasons for vaccine refusal were insufficient information (36.8%) and fear of side effects (19%). Correlates of positive vaccination intention were knowledge about HPV (OR = 1.02; 99% CI = 1.01–1.02). Conclusions: The findings suggest that the Greek government’s continuing HPV promotion efforts and education on the risks of HPV infection among young people are likely to increase vaccination acceptance among this group.

## 1. Introduction

Human papilloma virus (HPV) is one of the most common sexually transmitted infections, with an estimated 80% of people being infected with HPV in their lifetime [1]. HPV is widely known as the main causative agent for cervical cancer. Multiple HPV types are capable of infecting the anogenital tract, although only the high-risk types of HPV are oncogenic. The two most common are HPV-16 and -18, which, between them, are detected in 60–78% of cervical carcinomas and 72–94% of adenocarcinomas [2]. In Greece, HPV prevalence ranges between 5.2 and 6.7% and in Europe between 3.7 and 3.9% [3]. About 5–10% of all infected women develop persistent infection, which may progress to premalignant and malignant conditions [4]. The potential exists, however, to prevent the majority of these cancers through increased awareness programs leading to higher HPV vaccine uptake.

There are today three licensed prophylactic vaccines against HPV, exhibiting an excellent safety and immunogenicity profile. The quadrivalent vaccine (targeting HPV types 6, 11, 16 and 18) was first licensed in 2006, the bivalent vaccine (targeting HPV types 16 and 18) in 2007, and the 9-valent vaccine (targeting HPV types 6, 11, 16, 18, 31, 33, 45, 52 and 58) in 2014 [5]. By March 2017, globally, 71 countries had introduced the HPV vaccine into their national immunization program for girls and 11 countries also for boys. The World Health Organization (WHO) recommends the vaccination of young girls 9–13 years old, i.e., before the onset of sexual activity [4].

In Greece, since the introduction date in 2008, all vaccines have been fully financed by the national authorities and are offered to females 11–18 years old and to young men and women 18–26 years old who belong to special risk groups, according to the Greek National Immunization Programme (NIP) [6]. However, a number of Greek studies indicate low vaccination rates [7,8,9]. This low level of coverage has been attributed to the fact that HPV vaccination is considered the responsibility of the parents, there being as yet no national school-based program [10].

In order to achieve high vaccination coverage, it is necessary to obtain an overview of the knowledge about and attitude toward HPV vaccination in the general population. Previous studies have revealed that despite the high prevalence of HPV, there are misconceptions or else a lack of awareness about HPV even among otherwise well-informed people while, by contrast, there is evidence that vaccination uptake is increased when the targeted population is well-informed about the risks and benefits [11,12,13]. Thus, the present study aimed at assessing the extent of knowledge about, awareness of, and attitude toward HPV and its vaccine in a Greek population of young adults. We hypothesized that young adults who were considered vaccine-eligible (aged 25 years or younger) would have higher awareness and knowledge of HPV and HPV vaccination compared to older ages (i.e., somewhat older than 35 years). Similarly, we hypothesized that parameters such as the parents’ educational background, presymptomatic screening, and safe sexual behavior would influence vaccination acceptance among participants.

### Aim

The aim of this study was to investigate the perceptions, knowledge, and attitudes of young Greek adults concerning the prevention of HPV infection and HPV immunization.

## 2. Methods

### 2.1. Study Design

This study is based on a cross-sectional online survey.

### 2.2. Sample

A convenience sample was used to implement the present study. The study sample consisted of young Greek adults aged 17 to more than 35 years, living in Athens and urban areas. Participants who did not consent to participate in the study and those outside the study age range (under 17 years and over 40 years) were excluded. A total of 883 out of 926 people participated in the study. The inclusion criteria for participants were to be students attending medical schools and other higher education institutions and health professionals and be able to speak, read, and write in Greek. Exclusion criteria were inadequate language skills and age under 17 years.

### 2.3. Data Collection

The data collection was carried out through the completion of two anonymous questionnaires, after receiving informed consent from each participant. The questionnaires were converted into an online file via an electronic questionnaire creation platform and then posted in an electronic communication environment. Research data were collected from December 2020 to March 2021. Participation was voluntary and anonymity was assured. At the beginning of the research study, participants were asked to study the consent text, and only if they agreed would they complete the other sections. There was another consent question at the end of the questionnaire and answers were recorded only if the participants consented.

Before collecting data, we obtained approval by the Research Committee of the Hellenic Open University.

The research tools which evaluated the participants’ perceptions, knowledge, and attitudes concerning the prevention of HPV infection and HPV immunization were as follows:

A structured questionnaire indicated by Dafermou et al. [14] which included 67 questions and had five sub-dimensions, namely: demographic variables, knowledge of HPV, knowledge of the HPV vaccine, presymptomatic screening, and sexual behavior. The question items were open-ended and closed-ended, of which 10 items were addressed to female participants and the remainder were the same for both genders. This questionnaire had high reliability in Greek population [14].

A questionnaire indicated by Agorastos et al. [15] which included questions about personal history, knowledge about natural history and cervical-cancer prevention, HPV infection, its role in cervical malignancy, and the attitude of women toward HPV vaccination for themselves and their children. The above research tools were licensed with authorized permission for usage from the scientific authors. In addition, socio-demographic characteristics of the participants were collected.

### 2.4. Data Analysis

The Statistical Package for Social Sciences version 22.0 was used to perform the statistical analysis. Mean values and standard deviations (SD) were used to describe the quantitative variables. Absolute (N) and relative (%) frequencies were used to describe the qualitative variables. Pearson’s χ^2^ test or Fisher’s exact test was used to compare ratios where necessary. Student’s *t*-test was used to compare quantitative variables between two groups. Parametric dispersion analysis (ANOVA) was used to compare quantitative variables between more than two groups. To identify a type-I error due to multiple comparisons, Bonferroni correction was used, according to which the significance level was set at 0.05/k (k = number of comparisons). Linear regression analysis was used to find independent factors related to the knowledge scores, from which dependence coefficients (b) and their standard errors (standard errors = SE) were derived. In order to find independent factors related to vaccination and vaccination intention, a logistic regression analysis was performed, and the odds ratio (OR) was derived with 99% confidence intervals (99% DE). The internal reliability of the knowledge questionnaire was tested using Cronbach’s alpha coefficient. The significance levels were bilateral, and the statistical significance was set at 0.01.

## 3. Results

### 3.1. Participants’ Demographic Information

In total, 926 people participated in the survey, of whom 883 consented to participate; the response rate being 95.5% (Table 1). The mean age of the participants was 25.5 years (SD ± 8; range 17 to more than 35 years) and the majority (86.4%) (*n* = 763) were women. A total of 62.5% (*n* = 552) were students in higher education and 23.4% were full-time employees. Inhabitants of urban areas accounted for 73.5% of the study population and the remaining 24.6% lived in the capital city of Athens. In total, 863 participants (97.7%) were ethnic Greek. A total of 48.8% of respondents were unmarried, while 32.5% were in a relationship. In addition, 56.4% (*n* = 498) had a sibling and 46.5% had at least one parent with a higher-education qualification. Finally, 22.9% of the participants had a monthly family income of over EUR 2000.

### 3.2. Sexual Behavior and Screening

Most participants (81.1%) stated that they take precautions during sexual intercourse (Table 2). Males indicated a significantly higher rate of safe sexual behavior (90.8%), compared to females (80.3%) (*p* = 0.006). About 16.5% of respondents (*n* = 146) reported that they had been infected by HPV in the past, while male participants had a significantly lower HPV incidence rate compared to women (13.3% vs. 17%) (*p* < 0.001). The highest HPV infection rate according to age was found in the 26–35 age group (31.1%) (*p* < 0.001).

Approximately 57.2% of female participants (*n* = 431) had visited a gynecologist at least once in the past year. The frequency of visits to the gynecologist was found to be highest in the age group 26–35 years old (72.9%) and the lowest in the age group under 25 years old (22.0%) (*p* < 0.001). The majority (98.8%) knew about the Pap test, having been informed mainly by their family and the doctor (72.8 and 59.8%, respectively). It was found that, as participants’ age increased, the information rate via the gynecologist increased, while information via the Internet decreased (*p* < 0.001). Nearly 66.2% of the female population regularly had a Pap test, of whom 79.8% did it once a year, while 24% had been infected with HPV at some point in their lives. The Pap test rate differed significantly between the three age groups, with the lowest rate being in the age group of 25 years and under (50.4%) and the highest among women over 35 years (97.1%) (*p* < 0.001). Moreover, the rate of HPV infection among women who had cervical screening differed significantly between the age groups, with the lowest being in the group under 25 years of age (16.4%) and the highest in the 26–35 age group (29.9%, *p* < 0.001). The main reasons for not being screened were not being sexually active (62.5%), followed by the absence of symptoms (20.3%).

### 3.3. HPV Awareness

The majority of the study population was aware of HPV (90%) (Table 3). The mean HPV knowledge score was 53.26 out of 100 (SD ± 20.65). The internal consistency of the knowledge item regarding HPV evaluated by Cronbach’s alpha, was 0.77. Bivariate correlates of positive HPV awareness included the following: female sex (*p* = 0.001), employed (*p* = 0.001), married (*p* = 0.006), living in Athens (*p* = 0.001) and having previously been infected with HPV (*p* < 0.001). Moreover, there were significant differences in overall mean knowledge scores among the three age groups and the frequency of gynecologist visits. Participants who were 26–35 years old were more likely to report that they had heard of HPV, compared to those under 25 years (*p* = 0.001). Female participants who had cervical screening, visited the doctor regularly, and knew about the Pap test and HPV-DNA test had a higher score, indicating more knowledge regarding HPV (*p* < 0.001). In the multivariable model, the following factors were positively associated with HPV awareness: female sex (*p* = 0.007; referent = male sex), residence in the region of Athens (*p* = 0.002; referent = urban area) and positive HPV infection (*p* < 0.001; referent = negative infection). Parents’ educational level and safe sexual behavior were not associated with HPV awareness.

### 3.4. HPV Vaccination Awareness

Similarly, most respondents (88.6%) were aware of HPV vaccination. The mean HPV vaccine knowledge score was 38.92 out of 100 (SD ± 17.58). Internal consistency regarding the HPV-vaccine-knowledge item was 0.80. The most common information resources reported concerning the vaccine were the gynecologist (50.6%), followed by the Internet (43.6%) and the family (39.9%). Bivariate correlations of positive HPV vaccine awareness included the following: female sex (*p* < 0.001), married (*p* = 0.009), and women who had cervical screening and knew about the HPV-DNA test (*p* < 0.001). Participants 26–35 years old were better informed about the HPV vaccine than those over 35 years (*p* = 0.002). In the multivariable model, the factor of female sex was positively associated with vaccine knowledge (*p* < 0.001; referent = male sex). Occupation, residence, parents’ educational level, sexual behavior and HPV prevalence were not associated with HPV vaccine awareness.

### 3.5. Vaccination Uptake

Of the participants, almost 52.3% (*n* = 462) had already been vaccinated. Approximately 50.3% did not know which vaccine type they had received, while 60.9% had received all three HPV injections. Of those who had refused, a total of 36.8% reported lack of information as the main reason, followed by the fear of side effects (19%) and not being sexually active (15%). 

Bivariate correlates of HPV vaccination included the following: female sex, students, age up to 25 years, and HPV and HPV vaccination knowledge (*p* < 0.001) (Table 4). Vaccination uptake was lower among participants over 35 years (*p* < 0.001). Vaccinated participants knew significantly more about HPV and the vaccination (*p* < 0.001). In the logistic regression model, age, sex and the HPV-vaccine-knowledge score were positively associated with HPV acceptance. The results showed that the factors associated with higher vaccination rate in the entire sample were vaccinated participants being up to 25 years (OR = 0.07; 99% CI = 0.03–0.15; referent = participants up to 35 years), being female (OR = 11.99; 99% CI = 6.59–21.84; referent = male sex) and knowing better about the vaccine (OR = 1.04; 99% CI = 1.03–1.06). Screening factors for women, residence, marital status, parents’ educational background, sexual behavior and HPV infection were not associated with vaccination uptake, as had been hypothesized.

### 3.6. Vaccination Intention

Among the non-vaccinated respondents, 67.5% (*n* = 284) indicated that they would like to receive the vaccine if they were to decide in the future. According to bivariate correlation, the following factors significantly contributed to the participant’s vaccination intention: age, occupation, safe sexual behavior and greater knowledge about HPV (Table 5). The results showed that as age increases, the vaccination-intention rate decreases (*p* < 0.002). Students as well as those who take precautions during sexual intercourse had a significantly higher intention rate (*p* = 0.005). Moreover, participants who intended to get vaccinated knew better about HPV, compared to those who had no such intention (*p* = 0.001). Multiple logistic regression was performed to assess predictors of intention. The results indicated that the more participants knew about HPV, the more likely they were to have the intention to be vaccinated (OR = 1.22; 99% CI = 1.01–1.03). Screening for women, sex, the vaccine knowledge score and previous HPV infection did not appear to influence participants’ intention to be vaccinated.

### 3.7. Attitudes toward Vaccination

Among the total sample, 59.5% were willing to copay for the vaccine if it was not offered for free, while 26.2% indicated that they would pay only the participation rate in cost. The great majority (96.0%) stated that young people should be informed about the association between HPV with sexual behavior and cervical cancer before being vaccinated. Approximately 57.2% of participants believed that vaccination is beneficial for people who have already been infected by a specific HPV type, by offering protection from other HPV types.

## 4. Discussion

This study aimed to investigate the perceptions, knowledge, and attitudes of young Greek adults concerning the prevention of HPV infection and HPV immunization. The study results were encouraging, as they showed a favorable attitude toward vaccination and a moderate knowledge score for HPV and the vaccine. More than half of the study population had been vaccinated and about two out of three of the non-vaccinated were willing to receive the vaccine in the future.

The research findings revealed the moderate knowledge level of participants about HPV. Although the majority was aware of HPV, fewer than half knew of the possible transmission modes and only a quarter of the overall sample about the HPV–cancer association. To our knowledge, few similar studies on young adults have been conducted in Greece. One study on Greek students 15–18 years old observed limited knowledge about HPV [16], while another study on Greek females 17–24 years old found that 69.7% of them reported adequate knowledge about HPV [17]. On the other hand, a low knowledge level was also observed in several previous studies from other countries. A study in Romania revealed that 69.2% of women had heard about HPV, but their knowledge was minimal and incomplete [18]. Similarly, a study in China indicated that the participants had a poor understanding of HPV transmission [19]. Marek et al. [20] found that only 17% of Hungarian adolescents knew about HPV, while other studies reported a higher level of knowledge in this age group [21,22]. These findings suggest that, on the whole, participants did not have sufficient perceptions about HPV. Thus, there is an urgent need to raise public awareness about and increase education on the risks of HPV infection. In our study, as in other studies [23,24], female gender and marital status were found to be significantly related to knowledge level, as women and married participants proved to be more aware of HPV. This is possibly because, since females are more concerned about cervical cancer, they are better informed about HPV, and married people are more concerned about the perceived risk of HPV infection for themselves or for their daughter since they take most of the responsibility for their children’s healthcare. Furthermore, participants living in urban areas had a lower level of HPV knowledge, probably due to the fact that people living in large capital cities have greater access to health information sources. Varela and Saridi [25] showed similar results in their study. It was observed that people with a history of HPV know better about the transmission of HPV and the methods of protection. Focusing on age, the older age group proved to be more aware of HPV issues than the younger group. This is possibly due to the lack of information among the latter group as to HPV infection risks. Similar studies conducted in Nigerian and Canadian populations are in agreement with these findings [26,27].

Vaccination-related knowledge was at a lower level than HPV knowledge. More than three-quarters of respondents stated that they were aware of the HPV vaccine, but only one-third answered all the vaccine questions correctly. Similarly, a previous Greek study by Vaidakis et al. [28] found that less than half of the sample knew about the HPV vaccination. An international review of surveys on young people aged up to 26 years reported poor knowledge and misconceptions about HPV and its vaccine [29]. Furthermore, a meta-analysis of studies on European adolescents revealed a poor understanding of basic HPV vaccine knowledge [30]. Women were more knowledgeable about vaccination compared to men. These results are consistent with other previous studies, which showed that most females of the study population aged 18–26 years were more aware of the vaccine [31,32,33]. Women are probably more concerned about cervical cancer and want to be fully informed on prevention methods. Moreover, males have lower access to related information than women, who have more opportunities to acquire such information from healthcare professionals though cervical screening and routine gynecological visits. The younger participants received significantly lower scores than the older age groups, similarly to findings in previous research [34]. This is possibly explained by older people having attended more courses on sex education, knowing someone who has been diagnosed with HPV or experiencing infection themselves. Screening among female participants was positively associated with vaccine knowledge, as it was revealed that women who did not undergo Pap tests regularly had a lower knowledge level. Similar studies accord with these results [35,36]. This might be explained by the fact that HPV is statistically the main causative agent of cervical cancer, and it is detected in women only through presymptomatic screening. This accounts for HPV vaccination programs being targeted mostly at the female population [37].

Despite the relatively high level of awareness of HPV, only half of participants had been vaccinated. This rate is, nevertheless, well above the estimated national vaccination coverage of 9%. These results correspond to those of previous Greek research, which demonstrated above-average vaccination coverage [6,38,39]. Furthermore, consistent with the literature, our study results showed that young participants (<25 years old) and women were more likely to have been vaccinated [40,41,42]. This is probably due to the Greek vaccination program, which is targeted at young girls (12–18 years). Moreover, participants who were knowledgeable about HPV had higher acceptance of HPV vaccination. Evidence varies across countries as to whether knowledge impacts vaccination uptake. Some studies report a positive correlation [43,44], while others reported no correlation [45,46].

The study findings shed light on the need for the provision of more information on HPV, given that the main reason reported for vaccination reluctance was insufficient information, followed by fear of side effects. This can partly be attributed to misinformation spread by the Internet and the media, which leads to misconception about the safety and efficacy of the HPV vaccine. In fact, many international studies have determined that inadequate information about the vaccine and safety issues are common among both parents and adolescents [47,48,49].

Vaccination intention among non-vaccinated participants proved to be high, with more than half of participants stating that they were willing to be vaccinated in the future. Intention rate was higher among young participants, given that this age group (20–24 years old) are at the highest risk for contracting HPV, while older participants were less positive about vaccination, probably due to the perception of their low risk of HPV infection and being in a monogamous relationship. Moreover, people who followed safe sexual behavior were found to have a higher intention rate, the latter surely reflecting their awareness of and sensitivity to preventive measures. Xiao [50] demonstrated that sexual history is an important factor in predicting general behavior toward vaccination. Finally, participants who were knowledgeable about HPV were more likely to receive the vaccine in the future, this observation being in-line with other studies [14,51].

Regarding the source of information and trust, respondents stated that the gynecologist along with the Internet and family provided most of their information and were the most trusted. However, as mentioned, given that the Internet does not always provide accurate information, this finding suggests that governments must consider taking steps so that the Internet becomes part of a communication strategy that will promote the dissemination on the Web of reliable, scientifically evaluated information to young people and to the population at large. Meanwhile, healthcare providers can play a pivotal role in promoting vaccination, by cooperating with schools in providing health education to adolescents.

With respect to screening, two out of three female participants regularly had a Pap test, the majority of whom were over 35 years old. This is likely explained by the inclusion in the questionnaire of older women (over 35), who appear to have greater awareness of health issues and to routinely apply preventive behaviors. This finding is in-line with other Greek studies [52,53]. Participants aged 26–35 years old and especially women in this age group were more likely to be infected by HPV. This is probably due to the fact that these age groups take fewer sexual precautions, because they consider themselves to be at low infection risk. Moreover, women are more likely to be diagnosed with HPV through their routine gynecologist visits.

### Limitations

One limitation of the current study is its Web-based nature. Online surveys may have advantages related to the high speed and low cost of data collection. However, they may be biased by limited and selective participation, as certain populations with no access are unable to participate. Furthermore, using a convenience sample means that the sample is not representative of the general population so that there is the risk of bias. In terms of external validity, a larger sample size including more male participants as well as inhabitants of capital cities would render more valid results. One strength of the study is that the results may be used in the implementation of future research as well as in awareness campaigns concerning HPV prevention.

## 5. Conclusions

The aim of the present study was to investigate young Greek adults’ awareness of and attitudes toward HPV vaccination. In conclusion, the participants’ general attitude proved positive, as about half had been vaccinated and more than half were willing to receive the vaccine in the future. Vaccination uptake was significantly associated with knowledge of HPV and its vaccine, as well as other factors. The knowledge level was moderate and related to sex, residence, and previous HPV infection. The main vaccination barrier was inadequate information. Consequently, more educational programs are needed to raise awareness regarding HPV and the vaccination. These programs should be targeted both at health professionals and the general public. Future research studies need to collect and use data from a representative sample of the Greek population to provide a more in-depth evaluation of the views and predictors influencing HPV vaccination uptake in Greece.

## Figures and Tables

**Table 1:** Sociodemographic characteristics of participants.

		*n*	Valid %
Gender (*n* = 883)	Female	763	86.4
	Male	120	13.6
Age	Mean in years	883	25.5 (SD ± 8.0)
	Range in years	883	17–40
Ethnicity	Greek	863	97.7
	Albanian	12	1.4
	Other ^a^	8	0.9
Profession	Full-time employee	207	23.4
	Part-time employee	35	4.0
	Unemployed	42	4.8
	Student	552	62.5
	Household	7	0.8
	Do not know	6	0.5
	Student and Employee	34	3.9
Residence	Athens	217	24.6
	Urban area	649	73.5
	Foreign country	17	1.9
Marital status	Single	431	48.8
	Married	154	17.4
	Divorced	11	1.2
	In a relationship	287	32.5
Siblings	None	110	12.5
	One	498	56.4
	Two	183	20.7
	Three	62	7.0
	More	30	3.4
Parents’ Education	Primary education	138	15.6
	Secondary education	361	40.9
	Higher education	302	34.2
	Master’s	82	9.3
Monthly family income	EUR 500–1000	187	21.2
	EUR 1001–1500	155	17.6
	EUR 1501–2000	161	18.2
	Over EUR 2000	202	22.9
	Do not know	178	20.2

SD: standard deviation, ^a^ Other ethnicities were, in descending order: Cypriot (5), Bulgarian (1), Hungarian (1), Romanian (1).

**Table 2:** Sexual behavior and screening among participants.

	Total *n* (%)	Men *n* (%)	Women *n* (%)	*p* Pearson’s χ^2^ Test *	Age			*p* Pearson’s χ^2^ Test
					≤25	26–35	>35	
					*n* (%)	*n* (%)	*n* (%)	
Variables								
Sexual Behavior								
Precaution during sexual intercourse								
Yes	722 (81.8)	109 (90.8)	613 (80.3)		510 (86.9)	135 (76.3)	77 (64.7)	
No		11 (9.2)	150 (19.7)	**0.006**	77 (13.1)	42 (23.7)	42 (35.3)	0.310
HPV infection								
Yes	146 (16.5)	16 (13.3)	130 (17.0)		57 (9.7)	55 (31.1)	34 (28.6)	
No		104 (86.7)	633 (83.0)	**<0.001**	530 (90.3)	122 (68.9)	85 (71.4)	**<0.001**
Individual history of women								
Annual gynecology visit								
None			116 (15.0)		113 (22.0)	0 (0.0)	3 (2.9)	**<0.001**
Once			437 (57.2)		249(49.8)	116 (72.9)	72 (69.9)	
6 months			142 (18.8)		102 (20.7)	26 (16.8)	13 (12.6)	
Every 2 years			50 (6.6)		28 (5.7)	11 (7.1)	11 (10.7)	
>3 years			18 (2.4)		9 (1.8)	5 (3.2)	4 (3.9)	
Knowledge about cervical screening								
Yes			754 (98.8)		492 (98.2)	155 (100.0)	103 (100.0)	0.109 ^a^
No					9 (1.8)	0 (0.0)	0 (0.0)	
Information sources								
Family			549 (72.8)		377 (76.6)	116 (74.8)	52 (50.5)	**<0.001**
Friends			183 (24.3)		123 (25.0)	36 (23.2)	22 (21.4)	0.703
Television			102 (13.5)		66 (13.4)	16 (10.3)	19 (18.4)	0.173
Gynecologist			451 (59.8)		260 (25.8)	106 (68.4)	83 (80.6)	**<0.001**
Pediatrician			62 (8.2)		46 (9.3)	13 (8.4)	2 (1.9)	0.043
Press			54 (7.2)		26 (5.9)	14 (9.0)	9 (8.7)	0.301
Internet			293 (38.9)		212 (43.1)	51 (32.9)	28 (27.2)	**0.003**
School			195 (25.9)		139 (28.3)	33 (21.3)	22 (21.4)	0.120
Personal cervical screening								
Yes			502 (66.2)		252 (50.4)	150 (96.8)	100 (97.1)	**<0.001**
No			256 (33.8)		248 (49.6)	5 (3.2)	3 (2.9)	
Frequency of cervical screening								
Once a year			398 (79.8)		20 (80)	121 (81.2)	76 (76.8)	**0.011**
Every 6 months			34 (6.6)		23 (8.8)	9 (6.0)	2 (2.0)	
Every 2 years			53 (10.4)		26 (10.0)	14 (9.4)	13 (13.1)	
>3 years			17 (3.2)		3 (1.2)	6 (3.4)	8 (8.1)	
Reasons for not screening								
Ignorance			18 (7.0)		18 (7.3)	0 (0.0)	0 (0.0)	1.000 ^a^
Absence of sexual intercourse			160 (62.5)		154 (62.1)	4 (80.0)	2 (66.7)	0.847 ^a^
Absence of symptoms			52 (20.3)		51 (20.6)	1 (20.0)	0 (0.0)	1.000 ^a^
Fear of the result			10 (3.9)		9 (3.6)	0 (0.0)	1 (33.3)	0.125 ^a^
Difficult access			19 (7.4)		19 (7.7)	0 (0.0)	0 (0.0)	1.000 ^a^
Financial reasons			34 (13.3)		33 (13.3)	1 (20.0)	0 (0.0)	0.686 ^a^
Positive PAP Test for HPV ^b^								
Yes			145 (24.0)		71 (16.4)	46 (29.9)	28 (27.5)	
No			543 (76.0)		361 (83.6)	108 (70.1)	74 (72.5)	**<0.001**

* Pearson’s χ^2^ test < 0.01, ^a^ Fisher’ s exact test, ^b^ Concerns women who had cervical screening.

**Table 3:** Awareness of HPV and HPV vaccination among participants.

	Mean Knowledge Score % (SD)				Aware of HPV (*n* = 801; 90.7%)			Aware of HPV Vaccination (*n* = 782; 88.6%)		
	HPV 53.26 (20.65)	*p* Student’s *t*-Test *	HPV Vaccination 38.92 (17.58)	*p* Student’s *t*-Test	β ^a^	SE ^b^	*p* ** Value	β	SE	*p* Value
Variables										
Age										
≤25	51.83 (21.10)		38.59 (17.59)		(Referent)			(Referent)		
26–35	58.19 (18.67)		42.42 (16.33)		0.28	2.39	0.908	0.86	2.06	0.677
>35	53.00 (20.26)	**0.001**	35.37 (18.55)	**0.002**	−4.13	2.73	0.131	−5.98	2.36	0.011
Sex										
Male	47.32 (26.09)		32.92 (20.22)		(Referent)			(Referent)		
Female	54.19 (19.52)	**0.001**	39.87 (16.95)	**<0.001**	5.45	2.01	**0.007**	6.43	1.73	**<0.001**
Occupation										
Students	51.35 (21.30)		38.15 (17.42)		(Referent)			(Referent)		
Employed	57.08 (18.68)		40.40 (17.42)		4.40	2.32	0.058	2.52	2.00	0.209
Unemployed	57.29 (18.47)	**<0.001**	41.00 (18.54)	0.170	5.68	3.24	0.080	3.55	2.80	0.204
Residence										
Urban area	51.68 (21.29)		38.02 (17.77)		(Referent)			(Referent)		
Athens	57.41 (18.17)		41.20 (16.54)		4.92	1.59	**0.002**	3.03	1.37	0.027
Foreign country	60.50 (17.52)	**0.001**	44.12 (20.44)	0.033	6.18	5.06	0.222	5.27	4.36	0.227
Marital status										
Single	51.35 (21.54)		37.38 (17.88)		(Referent)			(Referent)		
Married	55.17 (19.56)	**0.006**	40.47 (17.15)	**0.009**	2.80	1.47	0.058	2.87	1.27	0.024
HPV infection										
No	51.60 (21.12)		38.41 (17.87)		(Referent)			(Referent)		
Yes	61.64 (15.67)	**<0.001**	41.50 (15.86)	0.050	8.47	1.90	**<0.001**	2.39	1.63	0.144
Data for women only:										
Annual gynecology visit										
None	47.60 (19.13)		35.76 (16.63)							
Once	55.55 (18.62)		41.25 (16.52)							
6 months	53.67 (19.73)		39.60 (16.17)							
Over 2–3 years	58.82 (23.03)	**<0.001**	41.18 (20.05)	0.020						
Personal cervical screening										
Yes	57.53 (18.63)		41.71 (17.01)							
No	47.56 (19.68)	**<0.001**	36.42 (16.27)	**<0.001**						
Knowledge about HPV–DNA Test										
Yes	64.66 (16.58)		47.44 (15.53)							
No	45.52 (17.53)	**<0.001**	33.84 (15.25)	**<0.001**						
Knowledge about cervical screening										
Yes	54.40 (19.38)		40.03 (16.92)							
No	30.95 (23.15)	**<0.001**	28.79 (13.64)	0.047						

SD: standard deviation, ^a^ dependency factor, ^b^ standard factor error, * *p* < 0.01, ** P Student’s *t*-test < 0.01.

**Table 4:** Multivariable logistic regression analysis for factors associated with vaccination uptake.

	Vaccinated			Logistic Regression OR (99% CI) ^a^		
	Yes *n* (%)	No *n* (%)	*p* Pearson’s χ^2^ Test *	OR	CI	*p* ** Value
Variables						
Age						
≤25	346 (58.9)	241 (41.1)	**<0.001**	(Referent)		
26–35	103 (58.2)	74 (41.8)		0.95	0.54–1.66	0.859
>35	13 (10.9)	106 (89.1)		0.07	0.03–0.15	**<0.001**
Sex						
Male	12 (12.5)	105 (87.5)	**<0.001**	(Referent)		
Female	447 (58.6)	316 (41.4)		11.99	6.59–21.84	**<0.001**
Ethnicity						
Other	12 (60.0)	8 (40.0)	0.487	(Referent)		
Greek	450 (52.1)	413 (47.9)		1.15	0.4–3.35	0.794
Occupation						
Students	334 (57.0)	252 (43.0)	**<0.001**	(Referent)		
Employed	101 (41.7)	141 (58.3)		0.82	0.47–1.44	0.483
Unemployed	25 (51.0)	24 (49.0)		0.86	0.39–1.87	0.701
Residence						
Urban area	347 (53.5)	302 (46.5)	0.372	(Referent)		
Athens	105 (48.4)	112 (51.6)		0.69	0.48–1.01	0.054
Foreign country	10 (58.8)	7 (41.2)		1.77	0.5–6.19	0.373
Marital status						
Single	230 (52.0)	212 (48.0)	0.845	(Referent)		
Married	232 (52.6)	209 (47.4)		1.09	0.77–1.53	0.623
Educational level of parents						
Primary	27 (40.9)	39 (59.1)	0.050	0.94	0.76–1.16	0.547
Secondary	153 (52.2)	140 (47.8)				
Higher	212 (51.6)	199 (48.8)				
Master’s	70 (61.90	43 (38.1)				
Safe sexual behavior						
No	72 (44.7)	89 (55.3)	0.033	(Referent)		
Yes	390 (54.0)	332 (46.0)		1.42	0.92–2.19	0.118
HPV infection						
No	393 (53.3)	344 (46.7)	0.180	(Referent)		
Yes	69 (47.3)	77 (52.7)		0.92	0.58–1.44	0.703
Mean HPV knowledge (SD)	56.4 (18.7)	49.9 (22.1)	**<0.001 ^b^**	1	0.99–1.01	0.584
Mean HPV vaccine knowledge (SD)	43.9(15.2)	33.4 (18.4)	**<0.001 ^b^**	1.04	1.03–1.06	**<0.001**

* Pearson’s χ^2^ test < 0.01, ** *p* < 0.01, ^a^ odds ratio (99% confidence interval), ^b^ Student’s *t*-test,.

**Table 5:** Multivariable logistic regression analysis for factors associated with vaccination intention.

	Intention to Vaccinate			Logistic Regression OR (99% CI) ^a^		
	Yes *n* (%)	No *n* (%)	*p* Pearson’s χ^2^ Test *	OR	CI	*p* ** Value
Variables						
Age						
≤25	179 (74.3)	62 (25.7)	**0.002**	(Referent)		
26–35	46 (62.2)	28 (37.8)		0.68	0.3–1.57	0.368
>35	59 (55.7)	47 (44.3)		0.58	0.26–1.29	0.180
Sex						
Male	73 (69.5)	32 (30.5)	0.602	(Referent)		
Female	211 (66.8)	105 (32.2)		0.97	0.57–1.64	0.899
Ethnicity						
Other	7 (87.5)	1 (12.5)	0.447 ^b^	(Referent)		
Greek	277 (67.1)	136 (32.9)		0.3	0.04–2.61	0.278
Occupation						
Students	186 (73.8)	66 (26.2)	**0.002**	(Referent)		
Employed	83 (58.9)	58 (41.1)		0.73	0.34–1.59	0.432
Unemployed	12 (50.0)	12 (50.0)		0.45	0.17–1.24	0.124
Residence						
Urban area	201 (66.6)	101 (33.4)	0.587 ^b^	(Referent)		
Athens	79 (70.5)	33 (29.5)		1	0.6–1.67	0.991
Foreign country	4 (57.1)	3 (42.9)		0.65	0.12–3.42	0.610
Marital status						
Single	153 (72.2)	59 (27.8)	0.038	(Referent)		
Married	131 (62.7)	78 (37.3)		0.92	0.55–1.52	0.739
Educational level of parents						
Primary	24 (61.5)	15 (38.5)	0.652	0.96	0.72–1.29	0.799
Secondary	91 (65.0)	49 ()35.0)				
Higher	139 (69.8)	60 (30.2)				
Master’s	30 (36.8)	13 (30.2)				
Safe sexual behavior						
No	49 (55.1)	40 (44.9)	**0.005**	(Referent)		
Yes	235 (70.8)	97 (29.2)		1.76	1.01–3.07	0.046
HPV infection						
No	229 (66.6)	115 (33.4)	0.411	(Referent)		
Yes	55 (71.4)	22 (28.6)		1.49	0.81–2.74	0.203
Mean HPV knowledge (SD)	52.2 (21.9)	44.9 (21.8)	**0.001 ^c^**	1.02	1.01–1.03	**0.004**
Mean HPV Vaccine knowledge (SD)	34.5 (18.6)	31.2 (17.8)	0.080 ^c^	1	0.98–1.01	0.651

* Pearson’s χ^2^ test < 0.01, ** *p* < 0.01, ^a^ odds ratio (99% confidence interval), ^b^ Fisher’s exact test, ^c^ Student’s *t*-test.

## Data Availability

The data presented in this study are available on request from the corresponding author. The data are not publicly available due to privacy reasons.
